# Insight into Steroid-Induced ONFH: The Molecular Mechanism and Function of Epigenetic Modification in Mesenchymal Stem Cells

**DOI:** 10.3390/biom14010004

**Published:** 2023-12-20

**Authors:** Chengxiong Huang, Liming Qing, Yu Xiao, Juyu Tang, Panfeng Wu

**Affiliations:** Department of Orthopedics, Hand and Microsurgery, National Clinical Research Center of Geriatric Disorders, Xiangya Hospital of Central South University, Changsha 410008, China; huangcxxyfy@163.com (C.H.); qingliming@csu.edu.cn (L.Q.); 218112335@csu.edu.cn (Y.X.)

**Keywords:** osteonecrosis of the femoral head, mesenchymal stem cells, epigenetic

## Abstract

Osteonecrosis of the femoral head (ONFH) is a common refractory orthopedic disease, which is one of the common causes of hip pain and dysfunction. ONFH has a very high disability rate, which is associated with a heavy burden to patients, families, and society. The pathogenesis of ONFH is not completely clear. At present, it is believed that it mainly includes coagulation dysfunction, abnormal lipid metabolism, an imbalance of osteogenic/adipogenic differentiation, and poor vascularization repair. The prevention and treatment of ONFH has always been a great challenge for clinical orthopedic surgeons. However, recent studies have emphasized that the use of mesenchymal stem cells (MSCs) to treat steroid-induced ONFH (SONFH) is a promising therapy. This review focuses on the role and molecular mechanism of epigenetic regulation in the progress of MSCs in the treatment of SONFH, and discusses the significance of the latest research in the treatment of SONFH from the perspective of epigenetics.

## 1. Introduction

Osteonecrosis of the femoral head (ONFH), also known as avascular necrosis of the femoral head, is one of the most common orthopedic refractory diseases [1]. As a result of insufficient blood supply, progressive osteocyte and bone marrow necrosis of the femoral head leads to structural changes and even collapse [2,3,4]. ONFH can be divided into traumatic and non-traumatic types [5]. The former occurs after physical trauma [6], while the etiology of non-traumatic ONFH is complex and multifactorial, mainly including long-term hormone therapy, excessive drinking, autoimmune diseases, and coagulation disorders [7,8]. Steroid-induced ONFH (SONFH) is the most common type of non-traumatic femoral head necrosis reported.

In different countries, the annual incidence of ONFH is about 1.5–3.0 per 100,000 of the population [9]. The age of ONFH patients is mostly 30–50 years old [10,11]. Improper treatment can lead to the loss of hip function in patients; most of these patients require hip arthroplasty [12]. However, the results of hip arthroplasty in young people are often not excellent and the failure rate is high due to various reasons such as prosthesis loosening, the excessive wear of polyethylene inserts, infection around the prosthesis, and short service life [13,14,15]. The early therapeutic intervention of ONFH is particularly important for preventing femoral head collapse, preserving joint function as much as possible, and avoiding hip replacement. Other joint preservation techniques, including physical intervention, drug therapy, osteotomy, and vascularized bone graft, remain unsatisfactory. ONFH brings great psychological pressure to patients and their families and lays a sizable burden on the social health care system. Unfortunately, the pathogenesis of ONFH has not been fully elucidated and treatment options are still limited.

With the development of regenerative medicine and tissue engineering technology, Hernigou first reported the treatment of ONFH with autologous bone marrow transplantation [16]. Stem cell transplantation is no longer limited to application in ischemic diseases. Furthermore, mesenchymal stem cells (MCSs) have shown encouraging results in animal experiments and clinical applications for the treatment of ONFH in recent years [17,18,19]. Bone marrow mesenchymal stem cells (BMSCs) are somatic stem cells with self-renewal and multi-directional differentiation potential which can differentiate into endothelial cells, osteoblasts, adipocytes, and chondrocytes [20]. Therefore, BMSCs play a crucial role in bone metabolism and tissue repair. By promoting BMSC proliferation, osteogenic differentiation is enhanced and lipogenic differentiation is inhibited.

In 1950, epigenetics was proposed by British developmental biologist Conrad Waddington [21]. He defined epigenetics as “the biological branch of studying the relationship between genes and their products to form phenotypes”, that is, all molecular pathways that regulate genotype expression to produce specific phenotypes [21]. Up util now, epigenetics has been broadly defined, primarily to refer to heritable variations that do not involve changes in the DNA sequence [22]. Epigenetic studies are generally divided into two categories: (1) Selective gene transcriptional regulation, including DNA methylation, DNA thiophosphorylation, histone modification, and chromatin remodeling. (2) Post-transcriptional gene regulation, including non-coding RNA (ncRNA) regulation, RNA modification, and nucleosome localization. Recent research has linked changes in epigenetic regulation to the development or progression of various human diseases. DNA demethylation and abnormal histone methylation play a key role in tumor metastasis and human cancer progression [23,24]. Similarly, other diseases are also negatively affected by epigenetic disorders, including diabetic nephropathy, osteoarthritis, and neurodegenerative diseases [23,25,26].

Recent studies suggest that the application of MSCs to enhance bone regeneration in patients with SONFH may be a promising therapeutic strategy [27,28,29]. Epigenetics refers to changing the phenotype of organisms by regulating gene expression without changing genetic material, and this change can be inherited. In other words, epigenetic regulation allows MSCs to maintain stable changes and can spread this effect through cell proliferation. Epigenetic regulation can be transmitted horizontally and vertically during stem cell proliferation or differentiation, thereby amplifying signals. Epigenetic regulation may be the cause of irreversible damage after hormone-induced ONFH withdrawal.

In order to apply epigenetic regulation to the treatment of SONFH, recent studies have shifted the focus to exploring the potential of combining MSCs with tissue engineering techniques. Lipid nanoparticles (LNPs) are currently the most promising clinical nucleic acid drug delivery carriers [30]. LNPs can prevent the degradation of nucleic acids in the blood circulation, and their specific components can achieve the efficient endosomal escape of nucleic acids [31,32]. Others based on biomaterials are being developed, including peptides, synthetic or natural-source polymers, and inorganic nanoparticles. These biomaterials are further processed into microspheres, hydrogels, and scaffolds to minimize cytotoxicity and enhance loading efficiency [33,34,35]. Focus has also shifted to exosomes, which may be a natural substitute for nanocarriers, that is, exosomes are “naturally domesticated nanocarriers” [36]. Studies have confirmed that MSC-Exos-miRNAs can reduce bone loss and improve SONFH.

In this review, we have summarized the latest research on epigenetic modification in the treatment of SONFH by mesenchymal stem cells, focusing on the molecular mechanism of epigenetic modification regulating the progress of SONFH. This is crucial for understanding the process of SONFH and is useful to guide the development of epigenetic regulators as direct or indirect targets for new drug treatment.

## 2. The Epigenetic Modification of SONFH

### 2.1. DNA Modification

DNA methylation is one of the forms of epigenetic modification. DNA methyltransferases (DNMTs) are involved in the long-term silencing of genes by transferring the methyl donor of S-adenosylmethionine (SAMe) to a specific base [37]. DNA methylation can occur at the N-6 position of adenine, the N-4 or C-5 position of cytosine, and the N-7 position of guanine [38]. In mammals, DNA methylation mainly occurs on the fifth carbon atom of cytosine, forming 5-methylcytosine (5 mC) [39]. CpGs in the human genome exist in the form of dispersed and highly aggregated CpG islands [40]. More than 80% of the CpG sites in the human genome are dispersed and highly methylated, while CpG islands are usually unmethylated and highly conserved. About 70% of the promoters contain CpG islands, and the methylation of CpG islands in the promoter region blocks the recognition and binding of transcription factors and participates in the regulation of gene expression throughout embryonic development [41].

**Figure 1:**
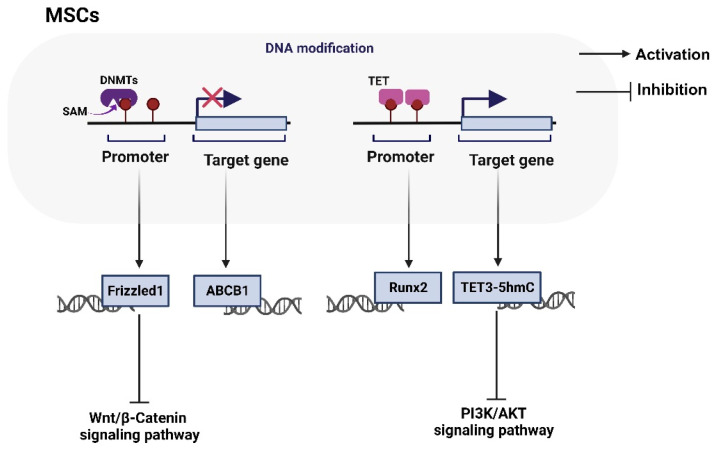
DNA modification of MSCs in SONFH. Under the action of DNA methyltransferases (DNMTs), S-adenosylmethionine (SAM) is used as a methyl donor to enhance gene methylation modification. Hypermethylation is considered to be an epigenetic marker of gene “silence”. During the progression of SONFH, the expression of Frizzled1 in mesenchymal cells is inhibited and Wnt/β-Catenin signaling pathway is upregulated and ABCB1 is downregulated. However, ten-eleven translocation (TET, DNA dioxidase) can gradually oxidize 5 mC (methylation of 5-methylcytosine) to 5 hmC (5-hydroxymethylcytosine). These advanced oxidation products cannot be recognized by DNMT1, thus hindering the maintenance of methylation patterns during DNA replication and indirectly promoting DNA demethylation. DNA demethylation in MSCs leads to inactivation of PI3K/AKT signaling pathway and promotes SONFH process.

A large number of studies have shown that reduced blood supply, osteogenic differentiation, and MSC cell proliferation and apoptosis are the main factors of SONFH. Here, we summarize the molecular mechanism (Table 1) and interaction model of the DNA methylation modification of MSCs in the progression of SONFH (Figure 1).

Wang et al. reported that low concentrations of TNFα promote osteogenic differentiation by activating the ephrinB2-EphB4 signaling pathway [47]. However, high concentrations of TNFα inhibit osteogenic differentiation by inhibiting the NF-kB signaling pathway [48] or activating Wnt/β-catenin signaling [49]. These studies did not further study the role of epigenetic regulation in the pathogenesis of SONFH. The research of Fang and Wu et al. filled this gap in knowledge [42,45]. During SONFH, TNFα in serum and bone marrow increases at the initial stage to promote MSC proliferation and angiogenesis. At the same time, TNFα mediates Runx2 (a specific transcription factor regulating the osteogenic differentiation of mesenchymal stem cells) methylation and inhibits its expression, thereby inhibiting osteoblast differentiation. Although TNFα plays a “double-edged sword” role in the pathogenesis of SONFH, on the one hand, it promotes MSC cell generation and angiogenesis, on the other hand, it maintains Runx2 methylation and inhibits osteoblast differentiation, but the interruption of blood circulation in the femoral head can lead to irreversible bone tissue necrosis [45].

Moreover, the aberrant hypermethylation of the CpG island of the FZD1 gene promoter in MSCs from SONFH patients leads to the inactivation of Wnt/β-catenin signaling and MSC cell dysfunction [42]. 5′-Aza-dC is an inhibitor of DNA methyltransferase (DNMT). 5′-Aza-dC treatment attenuates adipogenic differentiation and enhances osteogenic differentiation, which may be attributed to reversing the hypermethylation of the FZD1 promoter and activating the Wnt/β-catenin signaling pathway [42].

P-glycoprotein (P-gp) plays an important role in the absorption and distribution of drugs. Increased P-gp activity is a low-risk statistical marker for SONFH. The ABCB1 gene encodes P-gp. The hypermethylation of the ABCB1 gene promoter and the decrease of P-gp expression lead to the occurrence of SONFH when patients are treated with a glucocorticoid (GC) [44]. 5′-Aza-dC treatment can reverse the expression of ABCB1 in MSC, thereby alleviating the expression of P-gp and improving or delaying the dysfunction of MSC cells [44]. Moreover, as an important Chinese herbal medicine, Icariin induces the demethylation of the ABCB1 promoter and protects MSC from oxidative stress and lipogenesis in SONFH patients [43]. 5′-Aza-dC and Icariin can be used as potential drugs to target epigenetic changes for the treatment of GC-induced SONFH [43].

The above studies have shown that DNA methylation is involved in a variety of molecular mechanisms or signaling pathways in the process of SONFH. In addition, DNA methylation inhibitors showed positive therapeutic effects (such as 5′-Aza-dC and icariin). Methylation itself is a reversible chemical modification of DNA. It is promising to identify DNA methylation and change DNA methylation. A better understanding of the regulatory mechanism of DNA methylation in SONFH will facilitate the application of combination therapy and innovative drugs in the clinical treatment of SONFH patients.

### 2.2. Histone Acetylation and Histone Methylation

Chromatin remodeling is one of the effective ways to achieve epigenetic modification to study the interaction between environmental signals and genomes. Histones are small alkaline proteins, which are highly conserved in evolution. Histones were originally thought to be a packaging proteins responsible for compressing and assembling DNA, eventually forming the structural unit of the chromosome, the nucleosome [50]. In fact, the location and chemical modification of histones determine the structure and transcriptional activity of DNA, which in turn regulates gene expression [51]. Various modifications known to occur include acetylation, methylation, phosphorylation, citrullination, and ubiquitination [52]. Among them, the two most widely studied in the pathogenesis of SONFH are histone methylation and acetylation.

**Figure 2:**
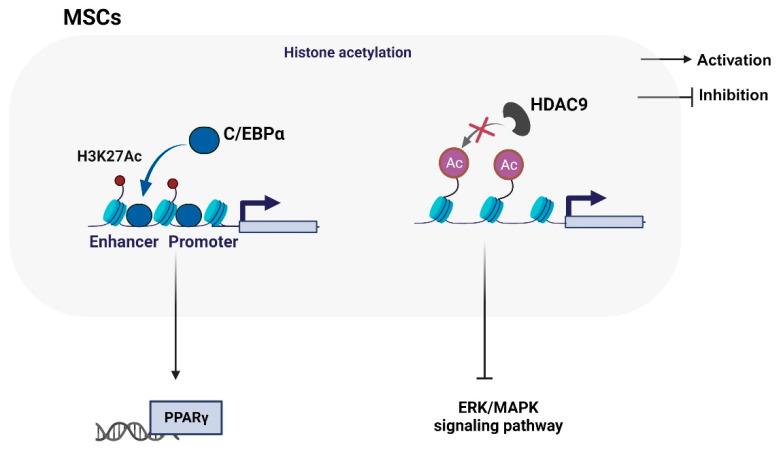
Histone acetylation modification of MSCs in the progression of SONFH. The transcription factor CCAAT/enhancer binding proteins alpha (C/EBPα) is recruited to the promoter of PPARΓ, promoting H3K27Ac modification and upregulating PPARγ expression. Moreover, the low expression of histone deacetylase 9 (HDAC9) inhibits histone acetylation modification and inactivates the ERK/MAPK signaling pathway, promoting the occurrence of SONFH.

Histone acetylation modification is generally associated with gene transcriptional activation, while histone deacetylation is associated with gene silencing. Some studies have reported that the histone acetylation modification of MSCs plays a key role in SONFH (Figure 2). The C/EBPs transcription factor family (C/EBPα, β, δ) regulates adipogenesis mainly by assisting in regulating the expression of adipocyte genes and affecting the uptake of glucose by adipocytes [19]. C/EBPα inhibits the osteogenic differentiation of BMSCs and promotes histone H3K27 acetylation (H3K27ac) in the PPARγ promoter region, mediating PPARγ activation and continuous expression [19]. Curcumin, as a typical histone acetylase inhibitor, reduces intramedullary adipogenesis in the femoral head by inhibiting the C/EBPα-mediated histone acetylation of PPARγ [19]. This study complements the specific molecular mechanism of PPARγ continuous expression leading to fat accumulation and SONFH.

Histone deacetylation is catalyzed by histone deacetylase (HDAC), recruited by transcription factors and protein complexes. The overexpression of HDAC leads to the enhanced deacetylation and enhanced positive charge of histones, thereby increasing the attraction between DNA and histones, making the relaxed nucleosomes very tight, which is not conducive to the expression of specific genes. The downregulation of HDAC9 expression inhibits the osteogenic differentiation of BMSC, partly by inhibiting the ERK/MAPK signaling pathway [53]. Notably, valproic acid (VPA), as an effective class I and class II HDAC inhibitor (HDACi), reduced the inhibitory effect of GC on the proliferation, apoptosis, and osteogenic differentiation of BMSC in vitro. In addition, VPA also retains the main blood supply of the femoral head of the experimental rats, which helps to prevent SONFH in rats [54].

Histone methylation not only has different modification sites, but also has different degrees of methylation of each residue, which greatly increases the complexity and diversity of histone methylation modification regulation. Although there are few studies on histone methylation in the pathogenesis of SONFH, more studies have focused on systemic administration to delay and prevent SONFH in existing studies. Neohesperidin (NH) is a compound extracted from citrus fruits. NH can improve the histopathological changes in steroid-induced SONFH mice, and this protective effect is achieved by regulating histone methylation modification [55]. Moreover, Huoxue Tongluo Capsule (HXTL Capsule) can also promote osteogenesis through histone methylation modification to ameliorate SONFH. These studies have provided sufficient evidence to explain the specific molecular mechanism [56]. It is worth developing drugs to prevent SONFH based on histone modification research.

### 2.3. Non-Coding RNAs (ncRNAs)

Non-coding RNA (ncRNA) refers to RNA that does not encode proteins [57,58]. Only 1–2% of the human genome encodes proteins, but up to 90% of the transcripts produced by the genome have no protein-coding ability. Functionally, ncRNAs are involved in various biological processes, such as cell proliferation, metabolism, and stem cell differentiation [59,60]. According to their biological functions, ncRNAs are divided into two categories: housekeeping and regulatory ncRNAs. Housekeeping ncRNAs (tRNA, rRNA, etc.) are widely expressed in cells and are necessary for cell survival [61]. Regulatory ncRNAs play an important role in epigenetic, transcriptional, and post-transcriptional levels by regulating gene expression. The three types of regulatory ncRNA are long non-coding RNA (lncRNA), circular RNA (circRNA), and microRNA (miRNA).

#### 2.3.1. LnRNAs/circRNAs in SONFH

LncRNA is defined as RNA with a length of more than 200 nucleotides and no coding function. In addition to these general characteristics of transcription and processing, lncRNA usually also contains embedded sequence motifs that can recruit certain nuclear factors, thereby promoting the nuclear localization and function of lncRNA. LncRNA located in the nucleus can interact with DNA, RNA, protein, and other molecules to regulate chromosome structure and function. A large proportion of lncRNAs are exported to the cytoplasm, and these lncRNAs may share the same processing and export pathways as messenger RNAs (mRNAs). Once localized in the cytoplasm, lncRNA usually trans-regulates gene expression at the post-transcriptional level, such as regulating mRNA translation and degradation, or participating in the regulation of intracellular signaling pathways.

CircRNA is another new type of endogenous ncRNA with a special covalent closure and single-stranded structure. CircRNA regulates gene expression and the coding of proteins. Although more and more circRNAs have been found in different species, only a few have been discovered and studied for their biological significance. In the past few years, people have become more and more interested in the function and pathogenesis of this special category of ncRNAs in SONFH. Here, we will briefly summarize the role of lncRNAs/circRNAs in the pathogenesis of SONFH to gain an in-depth understanding of its epigenetic function (Table 2). The epigenetic role of miRNAs in SONFH is described in the next section (Table 3).

**Table 2:** The role of lncRNAs/circRNAs in the pathogenesis of ONFH.

LncRNAs/circRNAs	Pathogenic Mechanism		Function	Type of Species	References
LncMALAT1		miR-329-5p/PRIP	Enhance osteoclast differentiation and inhibit osteoblast differentiation	GC-induced ONFH rat model	[62]
LncNORAD		miR-26a-5p	Enhance proliferation and osteogenic differentiation, inhibit apoptosis	Patients with SONFH	[18]
LNC00473		miR-23a-3p/PEBP1 miR-23a-3p/LRP5	Activate the Akt/Bad/Bcl-2 signaling pathway, enhance proliferation, and inhibit apoptosis Activate the Wnt/β-catenin signaling pathway, enhance osteoblast differentiation	Patients with GC-induced ONFH SONFH rat model	[63,64]
LncTmem235		miR-34a-3p/BIRC5	Inhibit hypoxia-induced apoptosis	SONFH rat model	[17]
LncFGD5-AS1		miR-296-5p/STAT3	Enhance proliferation, inhibit apoptosis, and OPG/RANK/RANKL signaling pathway	Patients with SONFH	[65]
CircUSP45		miR-127-5p/PTEN	Inhibit osteogenic differentiation	Patients with GC-induced ONFH	[66]
CircCDR1as	CeRNA	miR-7-5p/WNT5B	Inhibit osteoblast differentiation and enhance adipocyte differentiation	Patients with SONFH	[67]
CircPVT1		miR-21-5p/Smad3	Downregulate TGFβ/Smad2/3 expression, enhance osteoclast differentiation	SONFH rat model	[68]
Circ_0058792		miR-181a-5p/Smad7	Inhibit osteogenic differentiation and TGF-β signaling pathway	SONFH rat model	[69]
CircHGF		miR-25-3p/Smad7	Inhibit osteogenic differentiation and proliferation	Patients with SONFH	[70]
Circc_0058122		miR-7974/IGFBP5	Enhance apoptosis	Patients with SONFH	[71]
LncFAR591	Fos/Bim/Puma		Activate Bim and Puma-mediated mitochondrial apoptosis pathway.	SONFH rat model	[72]
LncMiat	HXTL capsule increased the occupancy of Miat promoter H3K27me3 and decreased the occupancy of H3K4me3.		Inhibit osteogenic differentiation	Patients with SONFH and rat bone marrow-derived MSCs	[56]
LncSNHG1	Neohesperidin drugs increased the occupancy of H3K27me3 and decreased the occupancy of H3K4me3 in the SNHG1 promoter.		Inhibit osteogenic differentiation	Patients with SONFH	[73]
Circ_0066523	Activate the PI3K/AKT pathway via recruiting KDM5B and inhibit PTEN expression.		Enhance proliferation and osteogenic differentiation	Human BMSCs	[74]

**Figure 3:**
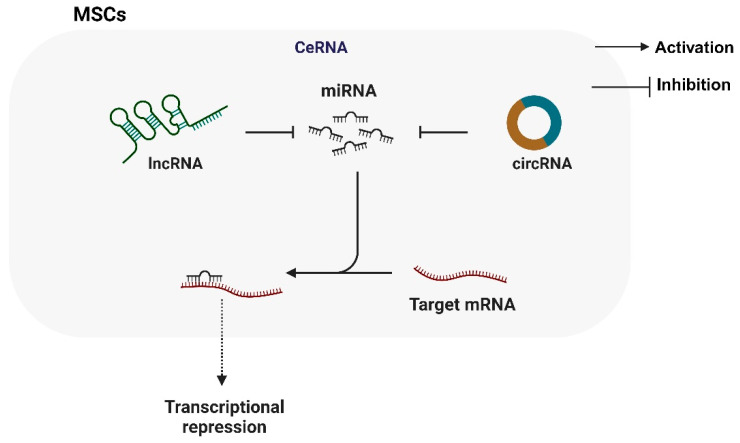
The regulation of CeRNAs mechanism on MSCs in SONFH.

Notably, most of the research focuses on the role of lncRNA/circRNA as competitive endogenous RNA (ceRNA). LncRNA/circRNA, such as ceRNA, prevents miRNA from inhibiting or degrading the translation of downstream target genes and participating in the regulation of MSCs (Figure 3). BMSCs are easily isolated and cultured, and their immunogenicity is weak. They are widely used in regenerative medicine. BMSC transplantation has been proven to be an ideal candidate for the early treatment of SONFH. Maintaining the survival of BMSCs in the osteonecrosis area is crucial to treatment. LncRNA Tmem235, as a ceRNA, competitively binds to miR-34a-3p with BIRC5 to prevent the inhibitory effect of miR-34a-3p on BIRC5 [17]. The activation of BIRC5 expression can effectively inhibit the hypoxia-induced apoptosis of BMSCs [17]. The hypoxic microenvironment in the femoral head necrosis area leads to a high apoptosis rate of transplanted BMSCs, which seriously limits the effect of osteogenic repair. However, LncTmem235, as a ceRNA, showed a reduction in the hypoxia-induced apoptosis of BMSC cells, which helped to improve the transplantation effect of BMSC transplantation on early SONFH [17].

Stem cell transplantation combined with tissue engineering has attracted considerable attention in the treatment of SONFH. LINC00473 upregulates PEBP1 by adsorbing miR-23a-3p, and activates the Wnt/β-catenin and PEBP1/Akt/Bad/Bcl-2 signaling pathways, which are involved in enhancing osteogenic differentiation, inhibiting adipogenesis and apoptosis [64]. Moreover, the co-transplantation of injectable thermosensitive polylactic-co-glycolic acid (PLGA) hydrogel loaded with LINC00473 could significantly attenuate the progression of SONFH [64]. PLGA hydrogel can help avoid the apoptosis, necrosis, and absorption of transplanted cells, which is a key factor for improving the survival of transplanted cells.

The ceRNA mechanism also plays a key role in the classical axis OPG/RANK/RANKL that regulates osteoblast–osteoclast bone homeostasis. LncNORAD and lncFGD5-AS1, as ceRNAs, target miR-26a-5p and miR-296-5p/STAT3, respectively, to regulate the major signaling pathways of osteoclast differentiation and bone resorption [18,65]. LncRNA/circRNA, as a ceRNA, is also involved in regulating other proliferation- and apoptosis-related pathways, including PI3K/Akt, Bad/Bcl-2, TGF-β, etc. [63,68,74].

In recent studies, lncRNA-mediated epigenetics have shown new regulatory mechanisms. A novel lncRNA was identified as Fos-related lincRNA ENSRNOT00000088059.1 (FAR591) [72]. Mechanistically, FAR591 is localized in the nucleus of (GC)-induced bone microvascular endothelial cells (BMECs) [72]. GC mediates the activation of glucocorticoid receptors and enters the nucleus. Glucocorticoid receptors act on the FAR591 gene promoter to activate FAR591 gene expression [72]. Further, FAR591 binds to the Fos gene promoter and recruits TATA-box binding protein-associated factor 15 (RNA-binding protein) and RNA polymerase II to mediate Fos transcriptional activation [72]. Fos ultimately activates mitochondrial apoptosis by regulating the Bim/Puma axis and mediates GC-induced BMEC apoptosis [72]. FAR591 leads to microcirculation dysfunction and the necrosis of the femoral head. The targeted inhibition of FAR591 may prevent and treat SONFH early.

#### 2.3.2. MiRNAs in ONFH

MiRNA is a class of small ncRNAs with a size of 17–25 nucleotides, which plays an important role in the post-transcriptional inhibition of mRNA in different eukaryotic lineages [61,75]. MiRNA binding to complementary target mRNA plays a central role in cell differentiation, proliferation and survival [76]. MiRNA is a rich non-coding RNA and a natural mechanism for organisms to achieve RNAi. MiRNAs are delivered to specific cells to promote the overexpression of beneficial genes and the silencing of harmful genes, thereby achieving the epigenetic regulation of functional genes [77]. Theoretically, gene therapy can achieve lasting or even curative effects. Later in 2019, nucleic acid delivery systems were developed rapidly in order to better prevent and treat pneumonia caused by COVID-19 infection [78,79,80]. In addition to antisense oligonucleotides (ASOs) and mRNAs, which have been extensively studied, miRNAs also have great potential for drug development due to their stability and prominent position in regulating gene expression. At present, the drug cobomarsen (miR-155 inhibitor), used in the treatment of cutaneous T-cell lymphoma, is in phase I clinical trials and is an orphan drug of the US FDA [81]. MRG-110 (miR-92 inhibitor) showed accelerated angiogenesis in a clinical model of heart failure [82]. Nevertheless, there is a lack of biomedical or clinical data on the role of miRNAs in human diseases.

The inherent limitations of miRNA hinder its application as a nucleic acid drug in clinical transformation, including its short half-life, sensitivity to enzymatic degradation, and limited membrane permeability. Consequently, improving the bioavailability of miRNA and changing the miRNA delivery strategy are crucial for achieving gene therapy. Recent studies have shown that miRNAs are present in exosomes (exos) during the osteogenic differentiation of BMSC. Stem cells can improve SONFH by exosome-mediated miRNA transport. All forms of organisms produce nanoscale extracellular vesicles called exosomes [83]. Exosomes themselves have biological activity, do not need more modification, and can be isolated from cells to play a therapeutic role. In addition, exosomes have stability, low immunogenicity and toxicity, and strong permeability. In recent years, exosomes have attracted attention as a new type of nucleic acid drug delivery carrier, which may be a promising alternative to nanoparticles [84,85].

**Figure 4:**
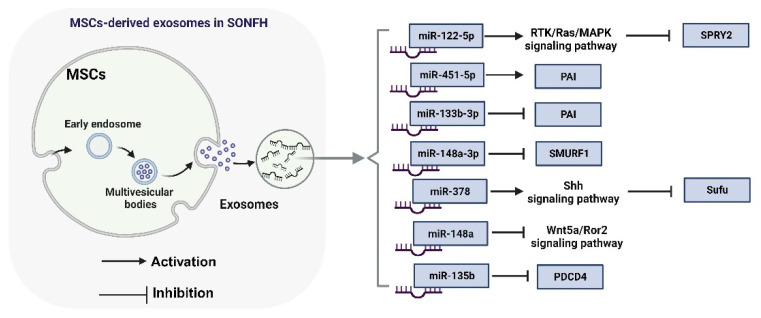
The biological process and molecular mechanism of MSC-derived exosomal miRNAs in the progression of SONFH.

Exosomes carry specific miRNA, which can enhance the osteogenic differentiation and angiogenesis of stem cells. Here, we summarize the molecular mechanism of miRNAs derived from MSCs in SONFH (Figure 4). It has been reported that exosomes rich in miR-122-5p can positively regulate the proliferation, osteogenic differentiation, and angiogenesis of BMSCs [86]. Mechanistically, BMSC-derived exosomes carry miR-122-5p to promote the repair and healing of SONFH by inhibiting SPRY2 expression and activating RTK/Ras/MAPK signaling pathway [86]. PAI-1-mediated hypofibrinolysis (reduced ability to cleave thrombus) leads to a microcirculation disorder of the femoral head, reduced blood flow of the femoral head, and ultimately leads to the ischemic necrosis of SONFH [87]. However, exosome miR-133b-3p from BMMSCs can inhibit PAI-1 expression and alleviate the loss of the fibrinolytic ability of vascular cells [87].

**Table 3:** Molecular mechanism and function of miRNAs in ONFH.

MiRNAs	Pathogenic Mechanism	Function	Type of Species	References
MiR-135b	FOXO1//Bim/Puma	Enhance proliferation, migration, and angiogenesis and inhibit apoptosis of endothelial cells	Patients with SONFH and SONFH rat model	[88]
MiR-148a-3p-MSC-Exos	SMURF1/SMAD7/BCL2	Enhance proliferation and osteogenic differentiation	ONFH rat model	[89]
MiR-27a-3p	PPARG	Enhance proliferation and osteogenic differentiation	Patients with SONFH and SONFH rat model	[90]
MiR-708	SMAD3	Inhibit osteogenic differentiation and adipogenic differentiation	Patients with SONFH	[91]
MiR-27a		Enhance proliferation, osteogenic differentiation, and TGF-β/SMAD7 signaling pathway	ONFH rat model	[92]
MiR-217	DKK1	Enhance proliferation and osteogenic differentiation	Patients with GC-induced ONFH	[93]
MiR-378-ASCs-Exos	Sufu/Shh	Enhance osteogenic differentiation and angiogenesis	ONFH rat model and human umbilical vein endothelial cells	[94]
MiR-133b-3p-BMMSC-Exos	PAI-1	Inhibit local fibrinolytic dysfunction	Nontraumatic osteonecrosis of the femoral head rabbit model	[87]
MiR-122-5p-BMSC-Exos	SPRY2	Enhance proliferation, osteogenic differentiation, angiogenesis, and activate RTK/Ras/MAPK signaling pathway	ONFH rabbit model	[86]
MVs-miR-148a	Wnt5a/Ror2	Enhance osteoclast differentiation and inhibit osteoblast differentiation	SONFH rat model	[95]
MiR-135b-MSC-Exos	PDCD	Inhibit apoptosis	Human BMSC and ONFH rat model	[96]
MiR-26a-CD43-Exos	SMAD3	Enhance osteogenic differentiation and angiogenesis	Human CD34 stem cell, human BMSC and SONFH rat model	[97]

## 3. Discussion

SONFH is a disabling joint disease. The treatment of patients with SONFH can no longer be satisfied by surgery alone [98]. It is very important to use drugs reasonably and effectively to strengthen the early prevention and treatment of SONFH and delay the development of the disease.

At present, the drugs used to treat ONFH are mainly divided into anticoagulants, antiplatelet drugs, vasodilators, and lipid-lowering drugs [99,100,101]. However, there is still a lack of specific drugs for the clinical treatment of SONFH. Emerging research has shown that the imbalance between osteoblast-mediated bone formation and osteoclast-mediated bone resorption and vascular damage are key factors in the pathogenesis of many SONFH cases. Therefore, drugs that effectively promote angiogenesis, such as ICA, pravastatin, and VO-OHpic, are potential SONFH therapeutic drugs [98,102,103,104,105]. In addition, compounds such as Valproic acid, Neohesperidin, and Huo Xue Tong Luo capsule can accelerate osteogenic differentiation and delay the progression of SONFH [106,107]. However, these compounds are still in the preclinical stage and are not sufficient for clinical application.

Furthermore, researches have shown that epigenetic regulation-mediated small molecule nucleic acids and inhibitors can also alleviate the progression of SONFH and show potential application value [18,64,73]. Because nucleic acid molecules are negatively charged and easily degraded by nucleases, the application of nucleic acid drugs is limited by in vivo delivery. The development of a reasonable nucleic acid delivery system and the safe and effective delivery of nucleic acid drugs to bone endothelial cells have become the key to the targeted therapy of SONFH. Exosomes and thermosensitive polylactic-co-glycolic acid (PLGA) hydrogel as nucleic acid delivery have shown great potential in the treatment of SONFH, but there are still some limitations that need to be overcome. The current technology is difficult to obtain pure exosomes and lacks specific markers for the successful extraction of exosomes. In addition, animal models can evaluate the short-term biocompatibility of hydrogels and cannot guarantee consistent long-term biocompatibility in clinical applications.

## 4. Conclusions

**Figure 5:**
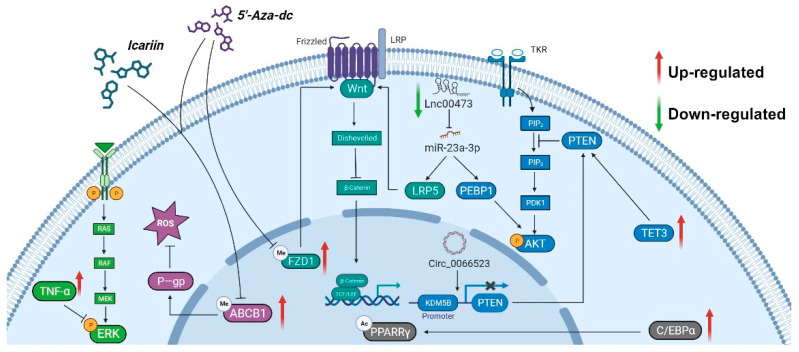
The molecular mechanism of epigenetic regulation in MSCs during SONFH progression.

In this review, we summarize the molecular mechanisms of the epigenetic regulation of MSCs in the progression of SONFH (Figure 5) and describe the key role of epigenetic regulation.

The molecular mechanism of the epigenetic regulation of SONFH development deserves further study, which is effective in guiding the development of epigenetic modulators as direct or indirect targets for new drug treatment. Finding suitable nucleic acid drug delivery carrier candidates is also a direction worthy of further study in the future. Moreover, the co-transplantation of bone endothelial cells and BMSCs may be a potential treatment method. The “best conditions” for the interaction between transplanted cells can be further studied, including improving the survival rate and cell number and proportion.

## Figures and Tables

**Table 1:** The DNA methylation sites and functions in ONFH.

Gene	State	Function	Type of Species	References
Frizzled1	Hypermethylation	Enhance osteogenic differentiation and inhibit adipogenic differentiation and Wnt/β-catenin signaling pathway	Patients with GC-induced ONFH	[42]
ABCB1	Hypermethylation	Enhance proliferation and osteogenic differentiation, inhibit ROS and adipogenesis	Patients with SONFH	[43,44]
Runx2	Hypermethylation	Inhibit osteogenic differentiation	Patients with SONFH and rat bone marrow-derived MSCs	[45]
TET3	Demethylation	Enhance osteocyte apoptosis and inhibit Akt signaling pathway	Patients with SONFH and SONFH rat model	[46]

## Data Availability

Data are contained within the article.
